# A CBCT Evaluation of Esthetic Preference Regarding the Perceived Facial Attractiveness of Young Korean Female Adults with a Normal Skeletal Pattern

**DOI:** 10.3390/s22197258

**Published:** 2022-09-25

**Authors:** Jin-Hyoung Oh, Jae Hyun Park, Heon Jae Cho, Hye Young Seo, Jong-Moon Chae

**Affiliations:** 1 Department of Orthodontics, School of Dentistry, University of Wonkwang, Iksan 54538, Korea; 2 Postgraduate Orthodontic Program, Arizona School of Dentistry & Oral Health, A.T. Still University, Mesa, AZ 85206, USA; 3 International Scholar, Graduate School of Dentistry, Kyung Hee University, Seoul 02447, Korea; 4 Dr. Cho’s Orthodontic Office, Seoul 06023, Korea; 5 Arthur A. Dugoni School of Dentistry, University of the Pacific, San Franciso, CA 94103, USA; 6 School of Big Data and Financial Statistics, College of Natural Sciences, University of Wonkwang, Iksan 54538, Korea; 7 Wonkwang Dental Research Institute, University of Wonkwang, Iksan 54538, Korea

**Keywords:** normal skeletal pattern, perceived facial attractiveness, esthetic preference, cone-beam computed tomography, three-dimensional cephalometric analysis

## Abstract

The aim of this study was to determine the factors that affect esthetic preference regarding the perceived facial attractiveness of young Korean female adults with a normal skeletal pattern using cone-beam computed tomography (CBCT). After reorienting the CBCT images of 40 young Korean female adults, three-dimensional (3D) measurements were analyzed, and five 3D facial images were digitally constructed. A computer-based questionnaire was used to determine perceived facial attractiveness by scoring from 1 (very unattractive) to 10 (very attractive). Esthetic perception and the most influential facial view and component were examined and compared by orthodontists, general dentists, and laypeople to evaluate facial attractiveness. Compared to the unattractive group, the attractive group had significantly greater values for Pog-NB, L1SI-FH, U lip thickness, L lip-U lip, and Nasolabial angle, along with significantly lower values for U1SI-FH and Cant of U lip; the attractive group also had a more retruded U1MP (*p* < 0.01), L1MP (*p* < 0.01), U lip (*p* < 0.05), and L lip (*p* < 0.01) relative to N, as well as a more retruded U lip (*p* < 0.05) and L lip (*p* <0.001) relative to esthetic lines. Orthodontists and general dentists were more likely to consider the profile view. When evaluating facial attractiveness, orthodontists and laypeople more often focused on the lips and chin. When evaluating facial attractiveness, all evaluators showed a high esthetic preference for retroclination and retrusion of the incisors and lips, but there were some differences in how they perceived facial attractiveness. Therefore, the ultimate perception of facial attractiveness needs to be based on the esthetic perceptions of laypeople.

## 1. Introduction

The goal of orthodontic treatment should be to create an ideal or functional occlusion and a well-balanced face. This raises a couple of questions, including “Do dental professionals and laypeople have the same perception of which facial esthetics look best?” and, if not, “Which perceptions, those of the professionals or patients, should prevail in treatment planning for orthodontic treatment?” [1,2,3,4,5,6,7,8,9,10,11,12,13,14,15,16,17,18,19,20].

Two-dimensional (2D) soft tissue facial or cephalometric analyses have been performed to aid in diagnosis and treatment planning to compare soft tissue profiles, optimize facial attractiveness, and investigate soft tissue adaptability to hard tissue [2,3,4,5,6,7,8,9,10,11,13,15,16,18,19,20]. Although these studies have improved the understanding of facial attractiveness, there was a vulnerability in their evaluation of facial attractiveness because it was investigated primarily in a profile view.

The application of three-dimensional (3D) imaging may give the clinician a more accurate evaluation of facial morphologies and therefore be useful for better understanding, comparing, and predicting treatment outcomes for orthodontic treatment or orthognathic surgery [12,21,22,23,24,25,26]. The advancement of 3D radiographs and their associated software programs has improved the ability to analyze both hard and soft tissue data. Three-dimensional cephalometric analyses provide information about not only the sagittal plane but also the intricate interrelationship between the sagittal, coronal, and axial dimensions of the craniofacial structure [24,25,26].

Several studies have used 3D imaging [11,12,21,22,23,24,25,26], but only a few focused on 3D facial esthetic preferences using 3D cephalometric analysis. Therefore, the purpose of this study was to evaluate the 3D facial attractiveness of young female adults with a normal skeletal pattern and then to investigate the differences in esthetic perception between orthodontists, general dentists, and laypeople. The null hypothesis states that there is no difference between the three groups of evaluators with respect to esthetic perception.

## 2. Materials and Methods

### 2.1. Subjects and Eligibility Criteria

This study was approved by the institutional review board (IRB) of Wonkwang University Daejeon Dental Hospital (W2202/003-001). All participants agreed to participate in this study and signed a written informed consent form.

The sample consisted of 40 young female adults (23.9 ± 4.7 years) who had cone-beam computed tomography (CBCT) scans taken for orthodontic treatment at Wonkwang University Daejeon Dental Hospital from January 2016 to December 2021. The CBCT (PSR 9000N; Asahi Alphard Vega, Kyoto, Japan) images were taken in C-mode (scan size, 200 × 179 mm; voxel size, 0.39 mm; field of view, 19.97 cm; scan time, 17 s; slice thickness, 1.0 mm) for pretreatment assessment. They had a normal skeletal pattern, according to the following inclusion criteria. These criteria were determined based on a previous study for the cephalometric analysis of normal Korean samples.

Inclusion criteria (3D normal skeletal criteria):

1. Sagittal: 0 mm < Pog(y) − A(y) < 4 mm
2. Vertical: 54% ≤ Lower facial height (A-Me)/Total facial height (N-Me) ≤ 57%
3. Transverse: −4 mm < Pog(x) < 4 mm

### 2.2. Standardized Reorientation of CBCT Images, 3D Coordinate System, and 3D Measurement

**Figure 1:**
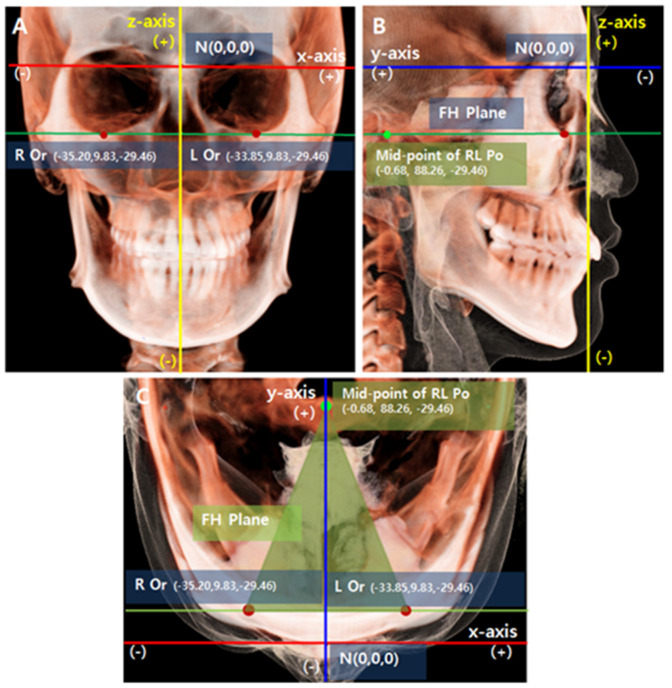
The coordinate system and standard reorientation of the CBCT Images. (**A**) Coronal view. (**B**) Sagittal view. (**C**) Axial view. The coordinate system consists of three axes (x, y, z) with their origin (0,0,0) registered at nasion (N). The Frankfort horizontal plane (FH plane) was constructed with bilateral orbitale (Or) points and the midpoint of bilateral porion (Po) points. The orbit line is a line passing both orbitale points in 3D space. The x-axis (red line) is a transverse axis passing through the N point and is also parallel to the FH plane in the coronal plane. The y-axis (blue line) is the anteroposterior axis passing through the N point and perpendicular to the x-axis. The y-axis is parallel to the FH plane in the sagittal plane. The z-axis (yellow line) is the vertical axis perpendicular to both the x- and y-axes. The x-axis is also parallel to the orbit line in the axial plane. Positive values are to the left, posterior, and superior (LPS) of the N point of the subject. Negative values are to the right, anterior, and inferior (RAI) of the N point.

ON3D (3DONS, INC., Seoul, Korea) software was used for this study. First, reorientation of the head position of each CBCT scan was performed to minimize any measurement errors from non-standard head postures [24]. The 3D coordinates (x, y, z) of all landmarks represent their 3D position relative to N (0,0,0) (Figure 1).

**Figure 2:**
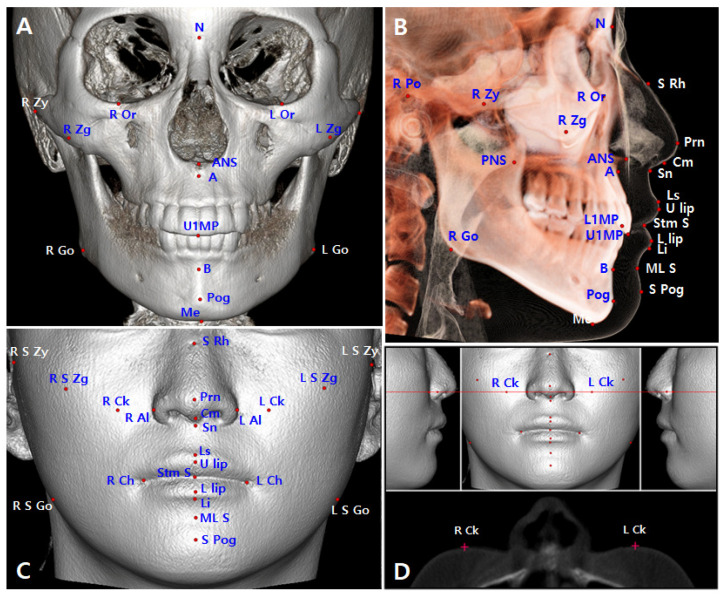
Three-dimensional landmarks. (**A**) Skeletal and dental landmarks in the coronal view. (**B**) Skeletal, dental, and soft tissue landmarks in the sagittal view. (**C**) Soft tissue landmarks in the coronal view. (**D**) Right cheek (R Ck) and left cheek (L Ck) points; the points located at the center of the cheek area and the most anterior point on the axial section of the red line (the red line passes through the median z value of the bilateral alar points and parallel to the x-axis). RL, right and left; N, nasion; Or, orbitale; Po, porion; Zy, zygion; Zg, zygoma; ANS, anterior nasal spine; PNS, posterior nasal spine; A, A point; B, B point; Pog, pogonion; Me, menton; Go, gonion; U1MP, the middle point of the maxillary central incisal tips; L1MP, the middle point of the mandibular central incisal tips; S Rh, soft tissue rhinion; Prn, pronasale; S Zy, soft tissue zygion; S Zg, soft tissue zygoma; Cm, columella; Sn, subnasale; Al, alar; Ck, cheek; Ls, labial superius; U lip, upper lip; Stm S, stomion superior; L lip, lower lip; Li, labial inferius; ML S, mentolabial sulcus; S Pog, soft tissue pogonion; S Go, soft tissue gonion.

**Figure 3:**
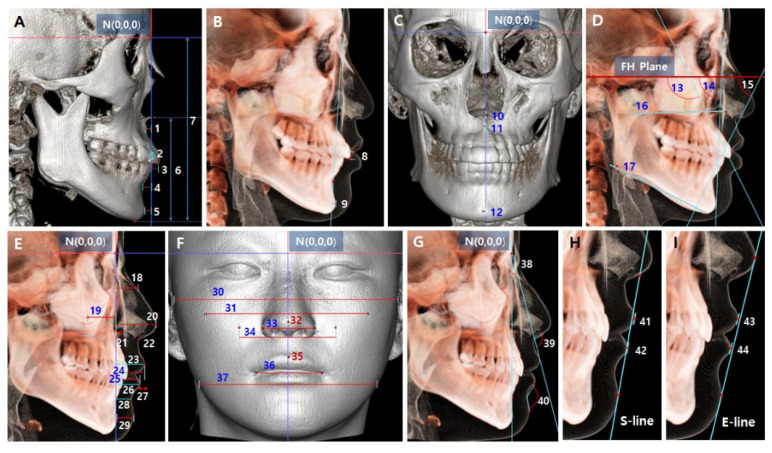
Three-dimensional values. (**A**,**B**) Skeletal and dental linear values in the sagittal view: 1, A(y); 2, L1MP(y); 3, U1MP(y); 4, B(y); 5, Pog(y); 6, LFH (lower facial height); 7, TFH (total facial height); 8, Maxillary incisal tip to Stm S; 9, Pog-NB. (**C**) Skeletal linear values in the coronal view: 10, ANS(x); 11, A(x); 12, Pog(x). (**D**) Skeletal and dental angular values in the sagittal view: 13, U1SI-FH (Upper incisor sagittal inclination to FH plane); 14, Facial line angle; 15, L1SI-FH (Lower incisor sagittal inclination to FH plane); 16, MxS line angle (Maxillary sagittal line angle); 17, MnS line angle (Mandibular sagittal line angle). (**E**) Soft tissue linear values in the sagittal view: 18, S Rh(y); 19, S Zg(y); 20, Prn(y); 21, S Ck(y); 22, Sn(y); 23, U lip(y); 24, U lip thickness; 25, L lip thickness; 26, L lip(y); 27, L lip(y)-U lip(y); 28, ML S(y); 29, Chin thickness. (**F**) Soft tissue linear values in the coronal view: 30, Midfacial width; 31, Bi S Zg; 32, Pronasale(x); 33, Nasal width; 34, Bi Ck; 35, Labial superius(x); 36, Mouth width; 37, Lower-facial width. (**G**) Soft tissue angular values in the sagittal view: 38, Cant of U lip; 39, Nasolabial angle; 40, Mentolabial angle. (**H**) Lip position relative to Steiner’s S-line: 41, U lip to S-line; 42, L lip to S-line. (**I**) Lip position relative to Ricketts’ E-line: 43, U lip to E-line; 44, L lip to E-line.

3D landmarks were automatically digitized with manual modification, and 3D measurements were made to determine the facial, skeletal, and dental characteristics of the 40 subjects using ON3D software (Figure 2 and Figure 3). Definitions of the 3D landmarks and values are described in Table 1A,B.

### 2.3. Evaluation of Perceived Facial Attractiveness Using a Questionnaire

**Figure 4:**
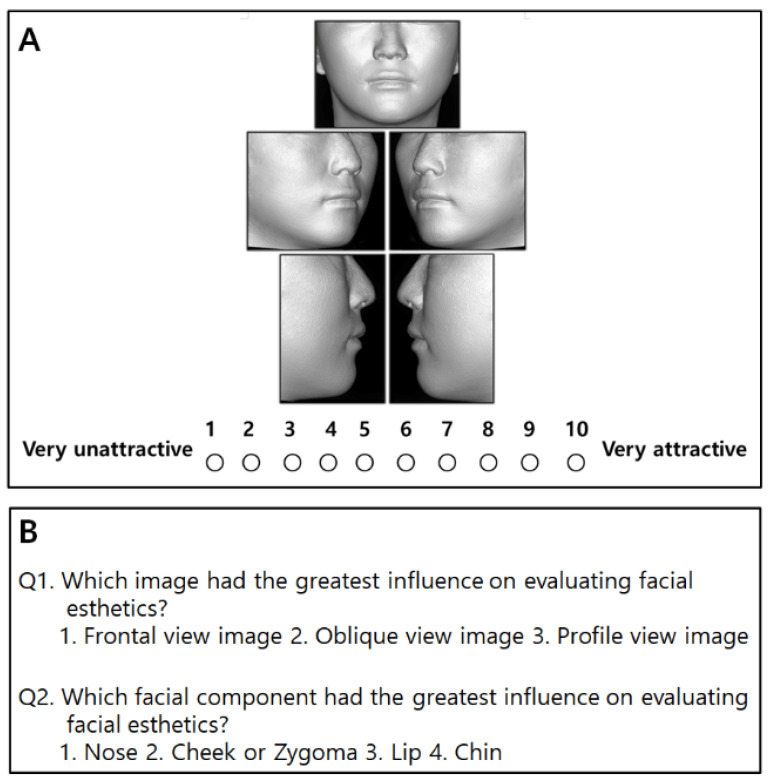
The questionnaire used to evaluate facial soft tissue esthetics. (**A**) Facial three-dimensional images and a numeric rating scale (NRS). (**B**) The two questions on the last page.

Images in soft tissue mode were captured from the frontal, oblique (left and right), and profile (left and right) views of 40 subjects using ON3D software. Thus, 3D soft tissue images consisting of a total of five photographs of each sample were constructed. Hair, eyes, and ears were excluded from the image to eliminate distractions, thus allowing evaluators to focus on the middle and lower face (Figure 4A).

The evaluators consisted of 42 orthodontists, 42 general dentists, and 42 laypeople. Data were collected via a computer-based questionnaire distributed personally to the participants by cell phone. The inclusion criteria for all evaluators included being at least 20 years of age and willing to participate in this study. The evaluators were asked to examine the facial 3D images for a sufficient time and then rank them from 1 (very unattractive) to 10 (very attractive) using a numeric rating scale (NRS) (Figure 4A). After that, they were asked the two questions shown on the last page of the questionnaire (Figure 4B).

According to the ranking of facial attractiveness, some of the 40 subjects were divided into either the attractive group (AG, top 20%, *n* = 8) or the unattractive group (UAG, bottom 20%, *n* = 8). Three-dimensional measurements were analyzed, and esthetic perception was compared between the three groups of evaluators. Additionally, it was determined which facial view was the most influential and which components were the most important in evaluating facial attractiveness.

### 2.4. Statistical Analysis

A power analysis using G*Power (version 3.1.9.7: Franz Faul, Christan-Albrechts-Universitat, Kiel, Germany) was performed to determine the sample size required for this study. It was determined that a total number of 126 subjects would provide a power of 0.87 and a two-tailed alpha value of 0.05.

To assess the reliability of the reorientation and digitizing process, CBCT scans of 10 subjects were re-digitized by the same operator after 3 weeks. Intraexaminer reliability was assessed by the intraclass correlation coefficient, which showed excellent reliability (ICC = 0.981~0.983). Statistical analysis was performed using SPSS software (version 27.0 for Windows; SPSS Corp., Chicago, IL, USA) for statistical analysis. An independent sample t-test was performed to determine the difference in 3D values according to perceived facial attractiveness between the AG and the UAG. Spearman’s rank correlation analysis was performed to determine the correlations between the ranks of the three evaluator groups. Cross-analysis was performed to determine the association between the evaluator groups for the image view and facial components when judging facial attractiveness. After the Shapiro–Wilk normality test was performed, if normality was not satisfied, a nonparametric test was performed. If the analysis of variance was significant, Scheffe’s post hoc test was performed. The significance level was 0.05.

## 3. Results

Compared to the UAG, the AG showed greater values for Pog-NB (*p* < 0.05), L1SI-FH (*p* < 0.01), U lip thickness (*p* < 0.05), L lip-U lip (*p* < 0.05), and Nasolabial angle (*p* < 0.001), and lower values for U1SI-FH (*p* < 0.001) and Cant of U lip (*p* < 0.001); the AG also exhibited a more retruded U1MP (*p* < 0.01), L1MP (*p* < 0.01), U lip (*p* < 0.05), L lip (*p* < 0.01), and ML S (*p* < 0.05) relative to N, as well as a more retruded U lip (*p* < 0.05) and L lip (*p* < 0.001) relative to esthetic lines (S-line and E-line). The 3D cephalometric results obtained from each of the groups of evaluators were almost the same as those obtained from the evaluators as a whole. The AG showed more retroclination and retrusion of incisors and lips than the UAG (Table 2A,B).

The most influential facial view (*p* < 0.05) and facial component (*p* < 0.001) with respect to perceived facial esthetics were significantly different between the three groups. Orthodontists (59.5%, *p* < 0.001) and general dentists (57.2%, *p* < 0.01) considered the profile view to be the most important, while laypeople (40.5%, *p* > 0.05) considered the frontal view the most important. Orthodontists (73.8%, *p* < 0.001) focused on lips, while general dentists (38.1%, *p* > 0.05) and laypeople (45.2%, *p* < 0.05) focused on the chin. The three groups of evaluators showed some differences in how they perceived facial attractiveness (Table 3).

## 4. Discussion

In this study, we evaluated facial attractiveness using 3D images obtained from CBCT and then derived 3D measurements and coordinates of faces that were perceived to be beautiful at the time of the study. Furthermore, we were able to acquire information about the normative mean values of the 3D coordinates and measurements of Korean female adults using data for the specific ethnic group [21,22].

Pog-NB and Pog(y)-B(y) values were greater in the AG than in the UAG in our study. This means that a more prominent Pog relative to the NB line and B(y) was considered to be more attractive; this is consistent with the results of a previous study [27]. On the other hand, Kambara et al. [27] concluded that a chin prominence (Pog-NB) greater than 4 mm could result in an unattractive face. From an esthetic standpoint, Pog-NB was related to lower lip position [6,7] and vertical skeletal pattern [28], which should be considered in order to identify the ideal lip position during treatment planning. Even so, in our study, the AG showed a more posterior position of Pog(y) relative to N than the UAG, which is consistent with the results of previous studies [1,2,3,21]. These results in young female adults may differ from those observed in young male adults [1,2,3], so further studies are necessary.

The positions of the tips of the cheeks and nose were not significantly different between the AG and the UAG in our study. However, some previous studies [12,26] suggested that maxillary skeletal expansion was correlated with forward movement of the cheek point and that cheek volume should be considered when evaluating facial attractiveness. Our study also showed that the lips were more posteriorly positioned in the AG than in the UAG, which means that somewhat retruded lips were perceived as more attractive. These results are consistent with those of previous studies [2,4] on the preference of facial esthetics in the Asian population. These studies reported that the public tends to prefer lips that are more retruded than average, although they have been distinguished by convex facial profiles.

In this study, significant differences were also found in U lip thickness and L lip(y)-U lip(y). Compared to the UAG, the AG showed greater upper lip thickness, which is consistent with some previous studies [11,29] that reported fuller lips were preferable. In addition, the L lip(y)-U lip(y) values were significantly greater in the AG. This indicates that it was considered more esthetic when the lower lip was positioned posteriorly relative to the upper lip. In addition, the preference for the lower lip position is closely related to the chin position [3,7].

Jang et al. [21] and Bayome et al. [23] reported that women chosen as Miss Korea had less chin and cheek volume than the general population. In our study, the attractive group tended to have a retrusive chin, but there were no significant differences in the values for the cheek or zygoma areas. The size and position of the cheeks and nose vary greatly from one individual to the next and should not be considered as a single factor that affects facial esthetics.

In this study, the most influential facial views and components used to judge facial esthetics were significantly different between the three evaluator groups, which is consistent with a previous study [30]. It is quite possible that esthetic perceptions are different among the evaluators depending on their training, educational background, and knowledge [2,8,15,17,20,30].

Yin et al. [2] suggested that orthodontists’ concepts of profile esthetics are influenced by their specialist training and preference for using cephalometric measurements to assess facial profiles. However, orthodontists should not forget that the ultimate perception of facial attractiveness should be based on the perceptions of laypeople and patients, not orthodontists [11,14]. Recognizing the differences in their esthetic perceptions may help orthodontists understand their patients’ expectations for orthodontic treatment and move them to consult with patients about the effects or limitations of treatment in terms of various facial areas during the diagnosis and treatment planning processes.

Smile esthetics, skin texture and color, lip color and form, etc., may affect the esthetic perception of facial attractiveness [31]. However, they were not included in this study because we could not take CBCT images that included them; this is a limitation of this study. Therefore, 3D photographs including these factors should be combined with CBCT images in future studies [32].

## 5. Conclusions

The null hypothesis was rejected; some differences were identified between the three groups of evaluators.

- When evaluating facial attractiveness, all three groups of evaluators showed a high esthetic preference for retroclination and retrusion of incisors and lips. Still, they showed differences in how they perceived facial attractiveness. The results are valid only in Asian populations, within the limitations of this study.
- The most influential facial views used to judge facial esthetics were the profile view for orthodontists (59.5%) and general dentists (57.2%) and the frontal view for laypeople (40.5%).
- The most influential facial components used to judge facial esthetics were the lips for orthodontists (73.8%) and the chin for general dentists (38.1%) and laypeople (45.2%).
- The ultimate perception of facial attractiveness needs to be based on the esthetic perceptions of laypeople.
- CBCT and an associated software program can be useful for evaluating facial esthetics by identifying the 3D positions of facial soft tissues.

## Figures and Tables

**Table 1:** (**A**). Definitions of three-dimensional landmarks. (**B**) Definitions of three-dimensional values.

(A)		
Landmarks	Definition	
**Cranial Base landmarks**		
N (Nasion)	The middle point of the frontonasal suture in the coronal plane	
RL Or (Orbitale)	The most inferior point of the orbital rim in the coronal plane	
RL Po (Porion)	The most superior point of the external auditory meatus	
**Maxillary landmarks**		
ANS (Anterior nasal spine)	The most anterior point of the premaxillary bone in the sagittal plane	
PNS (Posterior nasal spine)	The most posterior point of the palatine bone in the sagittal plane	
A (A point)	The deepest point in the anterior outline of the maxilla between the ANS and supradentale in the sagittal plane	
RL Zy (Zygion)	The most lateral point of the zygomatic arch, determined from the submento-vertex view	
RL Zg (Zygoma)	The point on the zygomatic bone at the turning point of bony curvature	
**Mandibular landmarks**		
B (B point)	The deepest point in the anterior outline of the mandible between infradentale and pogonion in the sagittal plane	
Pog (Pogonion)	The most anterior point in the mandibular chin area in the sagittal plane	
Me (Menton)	The lowermost point on the symphysis menti in the sagittal and coronal planes	
RL Go (Gonion)	The point in the inferoposterior outline of the mandible at which the surface turns from the inferior border into the posterior border in the sagittal plane	
**Dental landmarks**		
U1MP (Upper1midpoint)	Midpoint between both upper incisal tips	
L1MP (Lower1midpoint)	Midpoint between both lower incisal tips	
**Soft tissue landmarks**		
S Rh (Rhinion)	Soft tissue landmark in front of Rhinion (the lower end of the suture between the nasal bones)	
Prn (Pronasale)	The most prominent midpoint of the nasal tip	
Cm (Columella)	The most inferior and anterior point of the nose	
Sn (Subnasale)	Midpoint of the columellar base at the junction of the upper lip	
RL Al (Alar)	Most lateral point of the alar contour of the nose	
RL S Zy (Zygion)	The point on the facial skin lateral to the Zygion along the X-axis	
RL S Zg (Zygoma)	The point on the facial skin lateral to the Zygoma along the X-axis	
RL Ck (Cheek)	The point located at the center of the cheek area	
RL Ch (Cheillion)	Lateral extent of labial commissure	
Stm S (Stomion Superior)	Midpoint of the labial fissure between gently closed lips on the upper lip	
Ls (Labial superius)	The point that indicates the mucocutaneous limit of the upper lip in the sagittal plane	
Li (Labial inferius)	The point that indicates the mucocutaneous limit of the lower lip in the sagittal plane	
U lip (Upper lip)	The most anterior midpoint of the upper lip	
L lip (Lower lip)	The most anterior midpoint of the lower lip	
ML S (Mentolabial sulcus)	The deepest point in the anterior outline of the face between L lip and S Pog in the sagittal plane	
S Pog (Pogonion)	Most prominent midpoint of the chin	
S Go (Gonion)	The point on the facial skin lateral to the Gonion along the X-axis	
(**B**)		
**Values**	**Definition**	**View**
**Skeletal Values**		
(**1**) **Anteroposterior analysis**		
A(y) (mm)	y coordinate value of point A	sagittal
B(y) (mm)	y coordinate value of point B	sagittal
Pog(y) (mm)	y coordinate value of Pog	sagittal
B(y)-A(y) (mm)	Difference in y coordinate values between A and B	sagittal
Pog(y)-A(y) (mm)	Difference in y coordinate values between A and Pog	sagittal
B(y)-Pog(y) (mm)	Difference in y coordinate values between Pog and B	sagittal
Pog-NB (mm)	The perpendicular distance from Pog to N-B line	sagittal
Facial line angle (°)	Angle between the FH plane and N-Pog line	sagittal
SNA (°)	The angle between S, N, and A	sagittal
SNB (°)	The angle between S, N, and B	sagittal
ANB (°)	The angle between A, N, and B	sagittal
(**2**) **Vertical analysis**		
MxS line angle (°)	(Maxillary sagittal line angle) The degree of Maxilla (ANS-PNS) relative to the FH plane	sagittal
MnS line angle*(°)	(Mandibular sagittal line angle) The degree of Mandible (RL Go-Me) relative to the FH plane	sagittal
LFH (mm)	(Lower facial height) Vertical distance between ANS and Me	
TFH (mm)	(Total facial height) Vertical distance between N and Me	
LFH/TFH (%)	Ratio of Lower facial height divided by Total facial height	
(**3**) **Transverse analysis**		
ANS(x) (mm)	x coordinate value of ANS	coronal
A(x) (mm)	x coordinate value of A	coronal
Pog(x) (mm)	x coordinate value of Pog	coronal
**Dental values**		
U1MP(y)	y coordinate value of U1MP	sagittal
L1MP(y)	y coordinate value of L1MP	sagittal
L1MP(y)-U1MP(y)	Difference in y coordinate values between U1MP and L1MP	sagittal
B(y)-L1MP(y)	Difference in y coordinate values between L1MP and B	sagittal
U1SI-FH* (°)	Upper incisor sagittal inclination to the FH plane	sagittal
L1SI-FH* (°)	Lower incisor sagittal inclination to the FH plane	sagittal
U1MP to Stm S(mm)	Vertical distance between U1MP and Stm S	sagittal
**Soft tissue Values**		
S Rh(y) (mm)	y coordinate value of S Rhinion	sagittal
Prn(x) (mm)	x coordinate value of Pronasale	coronal
Prn(y) (mm)	y coordinate value of Pronasale	sagittal
S Zg(y)* (mm)	y coordinate value of S Zygoma	sagittal
Ck(y)*(mm)	y coordinate value of Cheek	sagittal
Sn(y) (mm)	y coordinate value of Sn	sagittal
Ls(x) (mm)	x coordinate value of Labial superius	coronal
U lip(y) (mm)	y coordinate value of U lip	sagittal
L lip(y) (mm)	y coordinate value of L lip	sagittal
L lip(y)-U lip(y)(mm)	Horizontal distance between U lip, L lip	sagittal
U lip to S-line (mm)	Steiner’s S-line (Columella-S Pogonion) to Upper lip	sagittal
L lip to S-line (mm)	Steiner’s S-line (Columella-S Pogonion) to Lower lip	sagittal
U lip to E-line (mm)	Ricketts’ E-line (Pronasale-S Pogonion) to Upper lip	sagittal
L lip to E-line (mm)	Ricketts’ E-line (Pronasale-S Pogonion) to Lower lip	sagittal
U lip thickness (mm)	(U1MP(y)-Upper lip(y)) Horizontal distance between U1MP and U lip	sagittal
L lip thickness (mm)	(L1MP(y)-Lower lip(y)) Horizontal distance between L1MP and L lip	sagittal
ML S(y) (mm)	y coordinate value of ML S	sagittal
S Pog(y) (mm)	y coordinate value of S Pog	sagittal
ML S(y)-Sn(y) (mm)	Difference in y coordinate values between Sn(y) and ML S(y)	sagittal
ML S(y)-S Pog(y) (mm)	Difference in y coordinate values between S Pog(y) and ML S(y)	sagittal
Chin thickness (mm)	(Pog(y)-S Pog(y)) Distance between Pog and S Pog	sagittal
Mid-facial width (mm)	Horizontal distance between right and left S Zy	coronal
Bi S Zg (mm)	Horizontal distance between right and left S Zg	coronal
Bi Ck (mm)	Horizontal distance between right and left Ck	coronal
Nasal width (mm)	Horizontal distance between right and left Al	coronal
Mouth width (mm)	Horizontal distance between right and left Ch	coronal
Lower-facial width (mm)	Horizontal distance between right and left S Go	coronal
LFW/MFW (%)	Ratio of Lower-facial width divided by Mid-facial width	coronal
Cant of U lip (°)	Angle between N-perpendicular and Sn-Ls line	sagittal
Nasolabial angle (°)	Angle between columella, Sn, and Ls	sagittal
Mentolabial angle (°)	Angle between Li, ML S, and S Pog	sagittal

FH, Frankfort horizontal plane. S, soft tissue; RL, right and left. * Average of right and left values.

**Table 2:** (**A**) Comparison of three-dimensional measurements of skeletal and dental values between the attractive group (AG) and the unattractive group (UAG), according to perceived facial attractiveness in total and for each group of evaluators. (**B**) Comparison of three-dimensional measurements of soft tissue values between the attractive group (AG) and the unattractive group (UAG) according to perceived facial attractiveness in total and for each group of evaluators.

(A)													
	Evaluators	Total			Orthodontists			General Dentists			Laypeople		
Measurement	All (*n* = 40)	AG (*n* = 8)	UAG (*n* = 8)	*p Value*	AG (*n* = 8)	UAG (*n* = 8)	*p Value*	AG (*n* = 8)	UAG (*n* = 8)	*p Value*	AG (*n* = 8)	UAG (*n* = 8)	*p Value*
**Skeletal Values**													
A(y) (mm)	−1.44 (2.39)	0.11 (2.45)	−1.92 (3.12)	0.170	−0.30 (2.77)	−2.78 (2.15)	0.066	0.14 (2.49)	−1.92 (3.12)	0.166	0.49 (2.24)	−1.92 (3.12)	0.097
B(y) (mm)	1.72 (2.79)	3.77 (3.06)	0.58 (3.60)	0.077	3.34 (3.41)	−0.16 (2.81)	0.042 *	3.28 (2.86)	0.58 (3.60)	0.119	4.12 (2.65)	0.58 (3.60)	0.042 *
Pog(y) (mm)	0.78 (2.68)	2.24 (3.06)	0.13 (2.78)	0.172	1.98 (3.37)	−0.46 (2.18)	0.107	1.95 (3.15)	0.13 (2.78)	0.242	2.42 (2.92)	0.13 (2.78)	0.131
B(y)-A(y) (mm)	3.16 (1.29)	3.66 (1.41)	2.50 (1.43)	0.124	3.64 (1.42)	2.61 (1.46)	0.176	3.14 (1.35)	2.50 (1.43)	0.370	3.63 (1.46)	2.50 (1.43)	0.140
Pog(y)-A(y) (mm)	2.23 (1.29)	2.13 (1.27)	2.06 (1.06)	0.898	2.28 (1.03)	2.32 (.94)	0.950	1.81 (1.42)	2.06 (1.06)	0.702	1.93 (1.44)	2.06 (1.06)	0.842
B(y)-Pog(y) (mm)	0.94 (1.12)	1.53 (.99)	0.45 (1.07)	0.054	1.36 (.76)	0.30 (.94)	0.026 *	1.33 (1.10)	0.45 (1.07)	0.124	1.70 (.82)	0.45 (1.07)	0.020 *
Pog-NB (mm)	1.15 (1.21)	2.06 (1.16)	0.44 (1.39)	0.024 *	1.83 (.99)	0.18 (1.06)	0.006 **	1.79 (1.19)	0.44 (1.39)	0.055	2.27 (.89)	0.44 (1.39)	0.007 **
Facial line angle (°)	89.52 (1.81)	88.85 (1.60)	89.98 (1.41)	0.155	88.99 (1.76)	90.27 (1.10)	0.102	89.00 (1.63)	89.98 (1.41)	0.220	88.75 (1.52)	89.98 (1.41)	0.117
SNA (°)	81.63 (2.59)	80.36 (2.23)	82.50 (3.77)	0.195	80.54 (2.41)	83.40 (2.93)	0.052	80.39 (2.48)	82.50 (3.77)	0.208	80.69 (2.25)	82.50 (3.77)	0.267
SNB (°)	79.24 (2.53)	78.21 (2.89)	80.30 (2.98)	0.178	78.25 (2.91)	80.79 (2.57)	0.086	78.56 (3.11)	80.30 (2.98)	0.273	78.72 (3.03)	80.30 (2.98)	0.310
ANB (°)	2.39 (1.19)	2.15 (1.18)	2.20 (1.40)	0.942	2.30 (1.23)	2.61 (1.14)	0.606	1.83 (1.30)	2.20 (1.40)	0.597	1.97 (1.28)	2.20 (1.40)	0.743
MxS line angle (°)	2.00 (1.31)	2.35 (1.54)	2.04 (1.01)	0.640	2.23 (1.64)	2.44 (1.19)	0.777	2.58 (1.39)	2.04 (1.01)	0.387	2.44 (1.51)	2.04 (1.01)	0.539
MnS line angle ^#^ (°)	25.19 (4.11)	25.13 (3.92)	26.69 (3.20)	0.398	24.49 (4.66)	26.14 (3.67)	0.443	25.69 (3.87)	26.69 (3.20)	0.582	24.33 (4.03)	26.69 (3.20)	0.215
LFH (mm)	65.31 (2.78)	64.32 (1.89)	65.95 (3.27)	0.242	64.35 (1.86)	65.31 (2.97)	0.450	63.95 (1.94)	65.95 (3.27)	0.160	63.97 (1.98)	65.95 (3.27)	0.165
TFH (mm)	118.10 (4.35)	116.96 (2.74)	118.78 (4.98)	0.382	116.89 (2.81)	117.68 (4.94)	0.699	116.21 (2.90)	118.78 (4.98)	0.228	116.43 (3.06)	118.78 (4.98)	0.275
LFH/TFH (%)	55.30 (.96)	54.98 (.79)	55.52 (1.03)	0.265	55.05 (.77)	55.50 (1.01)	0.329	55.03 (.78)	55.52 (1.03)	0.302	54.94 (.76)	55.52 (1.03)	0.223
ANS(x) (mm)	0.15 (1.08)	−0.56 (1.25)	0.20 (1.43)	0.280	−0.40 (1.26)	0.10 (1.44)	0.470	0.23 (1.04)	0.20 (1.43)	0.962	−0.41 (1.37)	0.20 (1.43)	0.398
A(x) (mm)	−0.27 (1.15)	−0.65 (1.28)	−0.25 (1.40)	0.559	−0.52 (1.31)	−0.46 (1.54)	0.936	0.15 (1.22)	−0.25 (1.40)	0.558	−0.47 (1.44)	−0.25 (1.40)	0.759
Pog(x) (mm)	0.14 (1.81)	−0.18 (1.75)	0.37 (1.58)	0.526	−0.24 (1.75)	0.25 (1.78)	0.593	0.49 (.97)	0.37 (1.58)	0.850	−0.23 (1.74)	0.37 (1.58)	0.486
**Dental values**													
U1MP(y)	−6.76 (3.67)	−3.20 (3.46)	−9.12 (3.71)	0.005 **	−3.90 (3.88)	−9.88 (3.18)	0.005 **	−4.00 (3.35)	−9.12 (3.71)	0.012 *	−3.21 (3.48)	−9.12 (3.71)	0.005 **
L1MP(y)	−3.77 (3.77)	−0.17 (3.69)	−6.22 (3.64)	0.005 **	−0.89 (4.04)	−7.18 (2.68)	0.003 **	−0.77 (3.70)	−6.22 (3.64)	0.010 *	−0.09 (3.60)	−6.22 (3.64)	0.004 **
L1MP(y)-U1MP(y)	2.99 (1.00)	3.03 (.44)	2.90 (1.34)	0.808	3.01 (.43)	2.71 (1.22)	0.519	3.23 (.49)	2.90 (1.34)	0.534	3.13 (.35)	2.90 (1.34)	0.655
B(y)-L1MP(y)	5.49 (2.23)	3.94 (1.38)	6.79 (2.01)	0.005 **	4.22 (1.21)	7.02 (2.04)	0.005 **	4.05 (1.48)	6.79 (2.01)	0.008 **	4.20 (1.49)	6.79 (2.01)	0.011 **
U1SI-FH ^#^ (°)	112.66 (5.48)	107.09 (4.39)	116.55 (3.74)	0.000 ***	107.86 (5.12)	117.15 (3.91)	0.001 **	109.80 (5.86)	116.55 (3.74)	0.016 *	108.91 (6.04)	116.55 (3.74)	0.009 **
L1SI-FH ^#^ (°)	63.41 (5.81)	66.85 (5.78)	58.38 (3.72)	0.004 **	66.23 (5.59)	57.48 (3.58)	0.002 **	67.74 (5.42)	58.38 (3.72)	0.001 **	66.84 (5.78)	58.38 (3.72)	0.004 **
U1MP to Stm S(mm)	2.70 (1.68)	2.36 (1.19)	2.99 (2.02)	0.464	2.94 (1.53)	2.50 (1.90)	0.612	1.97 (1.02)	2.99 (2.02)	0.227	2.04 (1.16)	2.99 (2.02)	0.269
**(B)**													
	**Evaluators**	**Total**	**Orthodontists**	**General Dentists**	**Laypeople**								
**Measurement**	**All (*n* = 40)**	**AG (*n* = 8)**	**UAG (*n* = 8)**	***p Value***	**AG (*n* = 8)**	**UAG (*n* = 8)**	***p Value***	**AG (*n* = 8)**	**UAG (*n* = 8)**	***p Value***	**AG (*n* = 8)**	**UAG (*n* = 8)**	***p Value***
**Soft Tissue Values**													
S Rh (y) (mm)	−14.05 (2.18)	−13.25 (2.41)	−13.90 (2.42)	0.596	−13.76 (2.34)	−14.39 (2.46)	0.606	−13.29(2.38)	−13.90(2.42)	0.617	−13.02(2.39)	−13.90(2.42)	0.474
Prn (x) (mm)	0.11 (1.25)	−0.05 (0.93)	−0.13 (1.63)	0.910	0.07 (0.98)	−0.36 (1.70)	0.546	0.15(0.98)	−0.13(1.63)	0.687	−0.17(1.01)	−0.13(1.63)	0.949
Prn (y) (mm)	−26.85 (2.71)	−25.34 (2.58)	−26.80 (3.21)	0.334	−25.88 (2.80)	−27.75 (2.41)	0.174	−25.41(2.53)	−26.80(3.21)	0.355	−25.11(2.61)	−26.80(3.21)	0.270
S Zg (y) ^#^ (mm)	3.79 (2.57)	4.58 (3.16)	3.29 (2.08)	0.351	4.32 (3.10)	2.82 (1.84)	0.259	4.43(3.23)	3.29(2.08)	0.414	4.93(2.81)	3.29(2.08)	0.205
Ck (y) ^#^ (mm)	−4.28 (2.05)	−3.55 (2.30)	−4.00 (2.33)	0.701	−3.63 (2.32)	−4.75 (2.06)	0.326	−3.56(2.30)	−4.00(2.33)	0.706	−3.19(2.19)	−4.00(2.33)	0.484
Sn (y) (mm)	−14.30 (2.72)	−13.03 (2.48)	−14.19 (3.44)	0.453	−13.40 (2.62)	−15.19 (2.93)	0.218	−13.47(2.14)	−14.19(3.44)	0.624	−12.86(2.36)	−14.19(3.44)	0.381
Ls (x) (mm)	0.11 (1.30)	−0.19 (1.01)	0.08 (1.84)	0.719	−0.14 (1.01)	−0.09 (1.84)	0.946	0.15(0.82)	0.08(1.84)	0.931	−0.21(1.00)	0.08(1.84)	0.699
U lip (y) (mm)	−18.85 (2.93)	−16.67 (2.79)	−20.02 (3.19)	0.042 *	−16.80 (2.91)	−20.61 (2.49)	0.014 *	−16.94 (2.79)	−20.02 (3.19)	0.059	−16.63 (2.77)	−20.02 (3.19)	0.040 *
L lip (y) (mm)	−16.77 (3.26)	−13.62 (3.15)	−18.92 (3.15)	0.005 **	−14.13 (3.65)	−19.64 (2.34)	0.003 **	−14.17 (2.85)	−18.92 (3.15)	0.007 **	−13.54 (3.10)	−18.92 (3.15)	0.004 **
L lip (y)-U lip(y)(mm)	1.98 (1.22)	2.63 (1.35)	1.08 (1.41)	0.041 *	2.68 (1.26)	0.94 (1.44)	0.022 *	2.36 (1.41)	1.08 (1.41)	0.092	2.69 (1.37)	1.08 (1.41)	0.037 *
U lip to S-line (mm)	0.72 (1.35)	0.49 (1.03)	1.94 (1.21)	0.022 *	0.21 (1.13)	1.79 (1.26)	0.019 *	0.41 (0.93)	1.94 (1.21)	0.013 *	0.56 (1.08)	1.94 (1.21)	0.030 *
L lip to S-line (mm)	1.45 (1.85)	0.40 (0.77)	3.71 (0.97)	0.000 ***	0.48 (0.75)	3.65 (1.01)	0.000 ***	0.67 (0.85)	3.71 (0.97)	0.000 ***	0.48 (0.80)	3.71 (0.97)	0.000 ***
U lip to E-line (mm)	−1.31 (1.32)	−1.57 (1.12)	−0.20 (1.20)	0.033*	−1.93 (1.10)	−0.31 (1.26)	0.016 *	−1.50 (1.28)	−0.20 (1.20)	0.055	−1.40 (1.31)	−0.20 (1.20)	0.075
L lip to E-line (mm)	0.26 (1.83)	−0.82 (0.79)	2.44 (1.02)	0.000 ***	−0.80 (0.79)	2.43 (1.03)	0.000 ***	−0.45 (0.83)	2.44 (1.02)	0.000 ***	−0.69 (0.86)	2.44 (1.02)	0.000 ***
U lip thickness (mm)	12.12 (1.62)	13.47 (1.52)	10.99 (1.81)	0.010 *	12.91 (1.43)	10.95 (1.82)	0.031 *	12.94 (1.65)	10.99 (1.81)	0.040 *	13.42 (1.57)	10.99 (1.81)	0.012 *
L lip thickness (mm)	13.01 (1.51)	13.45 (1.67)	12.75 (1.57)	0.401	13.24 (1.47)	12.52 (1.56)	0.360	13.40 (1.85)	12.75 (1.57)	0.464	13.45 (1.67)	12.75 (1.57)	0.402
ML S (y) (mm)	−9.91 (3.05)	−7.08 (3.17)	−11.02 (3.64)	0.037 *	−7.56 (3.56)	−11.69 (2.93)	0.024 *	−7.51 (2.95)	−11.02 (3.64)	0.052	−6.79 (2.97)	−11.02 (3.64)	0.023 *
S Pog (y) (mm)	−11.39 (3.16)	−8.98 (3.61)	−11.17 (3.16)	0.216	−9.37 (4.05)	−11.96 (2.58)	0.153	−9.24 (3.53)	−11.17 (3.16)	0.267	−8.72 (3.47)	−11.17 (3.16)	0.162
ML S (y)-Sn(y) (mm)	4.39 (1.96)	5.95 (1.19)	3.17 (2.40)	0.011 *	5.84 (1.35)	3.50 (2.64)	0.043 *	5.96 (1.12)	3.17 (2.40)	0.010 *	6.06 (1.14)	3.17 (2.40)	0.008 **
ML S (y)-S Pog(y) (mm)	1.48 (1.76)	1.90 (1.08)	0.15 (2.29)	0.072	1.80 (0.92)	0.27 (2.36)	0.110	1.73 (0.96)	0.15 (2.29)	0.094	1.93 (1.07)	0.15 (2.29)	0.066
Chin thickness (mm)	12.17 (1.59)	11.22 (1.40)	11.39 (1.73)	0.832	11.35 (1.45)	11.58 (1.77)	0.776	11.19 (1.43)	11.39 (1.73)	0.804	11.14 (1.43)	11.39 (1.73)	0.758
Midfacial width (mm)	147.31 (4.95)	147.78 (6.45)	145.94 (3.25)	0.483	148.83 (6.54)	146.23 (3.03)	0.326	146.46 (6.19)	145.94 (3.25)	0.837	147.92 (6.50)	145.94 (3.25)	0.455
Bi S Zg (mm)	105.55 (5.21)	103.08 (6.40)	103.69 (1.76	0.804	34.84 (0.98)	36.68 (3.00)	0.120	102.81 (6.20)	103.69 (1.76)	0.710	102.91 (6.27)	103.69 (1.76)	0.745
Bi Ck (mm)	63.37 (15.42)	65.84 (2.85)	67.97 (2.70)	0.146	103.73 (7.00)	104.06 (1.73)	0.900	65.57 (2.65)	67.97 (2.70)	0.094	65.83 (2.84)	67.97 (2.70)	0.145
Nasal width (mm)	36.16 (2.47)	35.15 (1.31)	36.20 (3.26)	0.413	65.94 (2.88)	67.62 (2.67)	0.247	35.19 (1.68)	36.20 (3.26)	0.453	34.99 (1.42)	36.20 (3.26)	0.355
Mouth width (mm)	45.36 (3.43)	44.09 (1.93)	43.35 (3.63)	0.618	44.04 (1.90)	45.24 (3.94)	0.452	44.32 (1.89)	43.35 (3.63)	0.514	44.36 (1.93)	43.35 (3.63)	0.498
Lower-facial width (mm)	118.70 (5.39)	117.59 (4.96)	117.87 (3.39)	0.894	118.40 (3.81)	118.98 (4.03)	0.768	115.86 (4.91)	117.87 (3.39)	0.356	117.15 (4.98)	117.87 (3.39)	0.740
LFW/MFW (%)	80.60 (3.01)	79.60 (2.28)	80.79 (2.46)	0.333	79.62 (2.25)	81.40 (3.07)	0.207	79.14 (2.40)	80.79 (2.46)	0.197	79.24 (2.52)	80.79 (2.46)	0.234
Cant of U lip (°)	17.99 (7.25)	13.44 (4.66)	24.57 (4.67)	0.000 ***	12.80 (3.74)	23.13 (6.73)	0.002 **	13.66 (4.94)	24.57 (4.67)	0.000 ***	13.80 (4.90)	24.57 (4.67)	0.000 ***
Nasolabial angle (°)	96.61 (10.23)	102.96 (3.32)	88.27 (8.39)	0.000 ***	104.02 (2.74)	90.19 (8.39)	0.000 ***	102.32 (4.29)	88.27 (8.39)	0.002 **	102.45 (3.48)	88.27 (8.39)	0.000 ***
Mentolabial angle (°)	129.62 (12.57)	128.39 (10.89)	129.84 (16.68)	0.839	129.56 (9.00)	128.34 (17.19)	0.861	129.78 (9.47)	129.84 (16.68)	0.992	127.35 (10.14)	129.84 (16.68)	0.723

Data are presented as means (standard deviation). ^#^ Average of right and left values. Independent *t*-tests were performed. * *p* < 0.05, ** *p* < 0.01, *** *p* < 0.001 (2-tailed).

**Table 3:** Perception of evaluators regarding image views and facial components on evaluating facial attractiveness.

	Orthodontists (*n* = 42)	General Dentists (*n* = 42)	Laypeople (*n* = 42)	*p* Value
**Facial views**				
Frontal	6 (14.3%)a	10 (23.8%)a	17 (40.5%)	0.01 *
Oblique	11 (26.2%)a	8 (19%)a	14 (33.3%)	
Profile	25 (59.5%)b	24 (57.2%)b	11 (26.2%)	
*p value*	0.000 ***	0.004 **	0.526	
**Facial components**				
Nose	2 (4.8%)a	5 (11.9%)	5 (11.9%)a	0.000 ***
Cheeks or Zygoma	2 (4.8%)a	10 (23.8%)	7 (16.7%)a	
Lips	31 (73.8%)b	11 (26.2%)	11 (26.2%)ab	
Chin	7 (16.6%)a	16 (38.1%)	19 (45.2%)b	
*p value*	0.000 ***	0.121	0.012*	

Data are presented as frequency (%). Chi-square tests were performed. The letters a < b indicate vertical differences that were analyzed by Scheffe’s homogeneous subset group (*p* <0.05). * *p* < 0.05, ** *p* < 0.01, *** *p* < 0.001 (two-tailed).

## Data Availability

The authors declare that the materials are available.
